# Kisspeptin and LH pulsatile temporal coupling in PCOS patients

**DOI:** 10.1007/s12020-018-1609-1

**Published:** 2018-05-04

**Authors:** Krzysztof Katulski, Agnieszka Podfigurna, Adam Czyzyk, Blazej Meczekalski, Alessandro D. Genazzani

**Affiliations:** 1 Department of Gynecological Endocrinology, Poznan University of Medical Sciences, Poznan, Poland; 2 Department of Obstetrics and Gynecology, Gynecological Endocrinology Center, University of Modena and Reggio Emilia, Modena, Italy

**Keywords:** Kisspeptin, PCOS, LH, Estradiol, Pulses

## Abstract

**Purpose:**

To evaluate the temporal coupling between spontaneous kisspeptin and luteinizing hormone (LH) pulsatile releases in polycystic ovary syndrome (PCOS) patients.

**Methods:**

We examined 71 patients diagnosed with PCOS. A 2 h pulsatility study was performed to evaluate serum kisspeptin and LH pulse frequency and concentration, sampled every 10 min; baseline follicle-stimulating hormone (FSH), estradiol (E2), prolactin (PRL), cortisol, 17-hydroksy-progesterone (17OHP), testosterone (T), free testosterone index (FTI, and insulin levels were also measured. Detect and Specific Concordance (SC) algorithms were used to evaluate the temporal coupling associations between spontaneous episodic secretion of kisspeptin and LH.

**Results:**

All PCOS patients demonstrated LH and kisspeptin pulsatile secretions. When the SC index was calculated across the sample of PCOS patients (*n* = 71), no temporal coupling was observed between kisspeptin and LH pulses. When PCOS patients were subdivided according to their menstrual cyclicity, oligomenorrheic patients demonstrated elevated kisspeptin pulse frequency. Additionally, the SC index reveled a temporal coupling between kisspeptin and LH secretory peaks only in eumenorrheic patients (*n* = 30, intermenstrual interval < 45 days). Oligomenorrheic PCOS patients (intermenstrual interval > 45 days) did not demonstrate temporal coupling between kisspeptin and LH secretory peaks.

**Conclusions:**

The study of the endogenous kisspeptin and LH pulsatile release revealed the temporal coupling of kisspeptin with LH secretory pulses only in eumenorrheic. This data supports the hypothesis that neuroendocrine impairments in PCOS affect the coupling of kisspeptin with LH pulses and potentially worsen as the disease progresses, becoming unequivocally evident in oligomenorrheic PCOS patients.

## Introduction

Identifying kisspeptin as a key mediator of gonadotropin-releasing hormone (GnRH) secretion has led to a new understanding of neuroendocrine regulation in human reproduction. Discovery of the kisspeptin-neurokinin-β-dynorphin (KNDy) pathway over recent years has further strengthened the scientific community’s understanding of the effects of endocrine, metabolic, and environmental inputs affecting GnRH secretion [1].

Kisspeptin plays a principal role in the regulation of gonadotropin secretion, the onset of puberty, sex hormone-mediated feedback, and adult fertility. Depending on the isoform, dose, and route of administration, kisspeptin has been shown to enhance secretion and pulse frequency of luteinizing hormone (LH) [1, 2]. Kisspeptin acts upstream to GnRH. Following paracrine stimulatory and inhibitory inputs from neurokinin B and dynorphin (KNDy neuropeptides), kisspeptin directly stimulates GnRH neurons to induce and modulate pulsatile GnRH release [3].

Human kisspeptin neurons are located primarily in two areas: the preoptic area and the hypothalamic arcuate nucleus (ARC), also known as the infundibular nucleus. Kisspeptin modulation of GnRH secretion depends on steroid sex hormone concentrations. In fact estradiol (E2) and prolactin (PRL) modulate kisspeptin activity both in the preoptic area and in the ARC receptors. In animal models preoptic kisspeptin neurons modulate periovulatory positive estrogen feedback on GnRH [4]. In a number of studies both preoptic and ARC neurons show a similar effect in humans [5]. It has been suggested that during the periovulatory period GnRH pulsatile release is influenced by kisspeptin’s regulatory effects on GnRH-secreting neurons. Kisspeptin-induced pulsatile GnRH secretion stimulates the release of both gonadotropins in an episodic manner from the pituitary [5]. The classic negative feedback due to estradiol during the follicular phase and to progesterone during the luteal phase is primarily exerted through specific effects on ARC kisspeptin neurons [5]. However, in humans not all GnRH-secreting neurons appear to display kisspeptin neuronal connections thus supporting the hypothesis that kisspeptin regulation of GnRH release from hypothalamic neurons is relatively more complex in humans [6–9].

Recently our research group at Poznan University of Medical Sciences and University of Modena demonstrated physiological coupling between kisspeptin and LH pulsatile secretions during the follicular phase of the menstrual cycle in healthy women [10]. Such coupling was revealed using sophisticated pulse detection and concordance algorithms analyzing spontaneous kisspeptin and LH pulsatile profiles. Our previous study demonstrated that kisspeptin and LH pulses are co-secreted, suggesting each kisspeptin pulse is accompanied by an LH pulse from the pituitary via a GnRH pulse induced from the hypothalamic GnRH-secretiong neurons [10].

Given the physiological effects of kisspeptin on GnRH-induced LH secretion, it is natural to postulate on the mechanism underlying neuroendocrine modulation exerted by kisspeptin in PCOS patients. It is commonly understood that PCOS is a complex multifactorial syndrome induced by abnormal neuroendocrine control and metabolic impairments [11]. Despite the vast body of literature on PCOS endocrinology and pathophysiology, no studies have focused on kisspeptin’s pulsatile secretion pattern and its correlation with episodic gonadotropin release in PCOS patients.

Increased secretions of LH, ovarian estrogen and androgen in patients with PCOS may be due to metabolic or hypothalamic-pituitary-ovarian axis abnormalitites or, as this study suggests, spontaneous kisspeptin release. The aim of this study is to investigate the association between spontaneous episodic secretion of kisspeptin and its temporal coupling with LH secretory pulses in a large sample size of PCOS patients.

## Materials and methods

### Study group

Seventy-one (*n* = 71) patients with PCOS aged 20–32 years old were selected from the Department of Gynecological Endocrinology Poznan University of Medical Sciences, Poznan, Poland; all participants provided informed consent. All women were Caucasian and admitted to the Department of Gynecological Endocrinology.

Diagnostic inclusion criteria for PCOS were in accordance with the American Society for Reproductive Medicine (ASRM) and the European Society for Human Reproduction and Embryology (ESHRE) consensus meeting. The diagnosis of PCOS was based on the association of at least two of the following: (a) oligomenorrhea with inter-menstrual intervals greater than 45 days, (b) clinical (acne, hirsutism) or biochemical signs of hyperandrogenism, (c) presence of polycystic ovaries via ultrasonography. Additionally, patients had to meet the following criteria: (d) absence of other endocrine disease including congenital adrenal hyperplasia (classical or non-classical), thyroid disorders, acromegaly or diabetes, (e) normal PRL levels (range 5–25 ng/ml), (f) no hormonal treatment for at least 6 months prior to the study. To try to maintain homogeneity of the group of patients recruited, only those with body mass index (BMI) above 25 kg/m^2^ were enrolled since overweight/obesity is a very frequent clinical characteristic in PCOS.

None of the subjects recruited were taking medications at the time of the study (e.g., steroid replacement therapy, oral contraceptives, metformin, or thiazide diuretics) or had used other drugs that could affect basal parameter status within 3 months of the evaluation. Pregnancy was excluded performing a urine hCG test.

The sample group was subdivided into two groups based on the length of the menstrual cycle: group A (*n* = 30) consisted of menstrual cycles of 45 days or less, and group B (*n* = 41) with menstrual cycles greater than 45 days with oligo/amenorrhea. The cycle length was estimated using self-reported menstrual calendars from the year prior to the study. The choice of such intermenstrual interval was arbitrary since oligomenorrhea is usually stated for intervals above 35 days. The presence of an abnormal intermenstrual interval of time was more selective on the side of oligomenorrheic PCOS patients, being these ones the target of the present study.

The local Ethics Committee of the Poznan University of Medical Sciences, Poland approved the study protocol. This observational study was not registered as a clinical trial as there was no intervention group.

Details of medical history emphasized menstrual details including age at menarche, regularity, duration, and number of cycles per year. Menstrual disturbances were classified as oligomenorrhea if there were less than eight cycles/year or menstrual intervals > 45 days, or amenorrhea if menses were absent for at least 3 months prior to the study. Other history included changes in weight, duration of infertility, family history of PCOS, family history of type 2 DM, illicit drug use, progression and distribution of hirsutism, and severity of and treatment response to acne vulgaris.

Anthropometric assessments included measurements of height (cm), body weight (kg), and calculation of body mass index (BMI). Additionally, blood pressure (BP) was measured. A detailed physical examination was performed on each participant, paying particular attention to grading of acanthosis nigricans, acne vulgaris, androgenic alopecia, and quantification of hirsutism. The modified Ferriman–Gallwey (FG) score was used to assess the degree of hirsutism with a score of eight being the minimum threshold to diagnose hirsutism out of a total of 36 from nine body areas.

Ultrasound examinations were performed on all patients. Ovarian volume was calculated using a simplified ellipsoid formula (0.5 × length × width × thickness). Ovarian volume was defined as the average volume of both ovaries, and the ovarian follicle number was defined as the average number of follicles within each ovary.

### Study protocol

All subjects participated in obtaining pulsatile measurements of LH and kisspeptin serum concentrations. The pulsatility study was performed during the mid-follicular phase of the menstrual cycle between days 6–10 from the onset of the last menstrual period. If patients were amenorrheic, the pulsatility study was done on a random day. To perform the pulsatility blood collection a heparinized well was placed in the antecubital vein 45–60 min before commencing venous sampling. Blood was withdrawn every 10 min for 2 h (from 09.00 to 11.10 A.M.) to measure LH and serum kisspeptin concentrations. The choice of this relatively long interval of time was done to minimize the blood withdrawal and assaying costs. Moreover, the sampling was similar to that used in a previous study (10). Pulsatility tests were scheduled in the morning (between 9:00 and 11:00) to minimize the confounding effects of environmental stressors related to daily activity. Patients were asked to fast on the morning of the study.

On the morning of the study, an additional blood sample was drawn to establish baseline serum concentrations of kisspeptin, luteinizing hormone (LH), follicle-stimulating hormone (FSH), E2, PRL, cortisol, dehydroepiandrosterone (DHEA), 17-ketosteroids (KS), testosterone (T), sex hormone binding protein (SHBG), glucose, and insulin. Blood samples used for kisspeptin assays were collected in EDTA tubes and centrifuged at 1600*g* for 15 min at 4 °C. For all other hormones, blood was allowed to clot before the serum was centrifuged at 1500*g* for 5 min. The serum was stored at −70 °C until the assays were performed.

### Assays

Kisspeptin was measured with the use of enzyme-linked immunosorbent assay (ELISA) and a Kiss-1 (112–121) Amide/Kisspeptin-10/Metastin (45–54) Amide (Human) EIA Kit 1 (Phoenix Pharmaceuticals). This ELISA kit provides 100% cross-reactivity with longer kisspeptin 1–54 forms, so it detects both forms of active kisspeptin, as previously described [10].

Kisspeptin samples from each subject were analyzed in the same assay. The interassay coefficient of variation for all hormonal assays was < 9% at the concentrations measured. Intra-assay coefficient of variation (CV) for LH was 6.8% with a sensitivity of 0.8 ng/mL, as previously described [10].

Serum FSH, LH, PRL, E2, and insulin serum concentrations were determined by means of electrochemoluminescence immunoassay (Roche Diagnostics). Concentrations of various serum hormones were measured with the use of a Cobas E601 analyzer (Roche Diagnostics). Intra and interassay %CV for all these assays were 4 and 7%, respectively. Insulin resistance was checked using the homeostasis model (HOMA-IR) with the use of the formula fasting glucose (mmol/L) × fasting insulin (mU/L)/22.5, and a HOMA-IR ≥ 2.5 threshold was interpreted as a measure of insulin resistance [12].

### Pulse detection and degree of concordance

#### Pulse detection

Time series of LH and kisspeptin were first evaluated separately to calculate the random measurement error on the duplicates of each time series using the program Predetec, a specific program of Detect software. Predetec calculates the mean (X) and variance (s2x) for each set of replicates in the time series. It plots s2x or sx versus X, or log(s2x) versus log(X) and then fits five different models to this relationship, selecting the best model in terms of the smallest root-mean-square error for sx, as previously described [10]. The program is subject to the constraint that the predicted sx cannot be negative for the observable range of the data. Finally, the program provides coefficients for the variance model to be used in the Detect program for pulse detection analysis [13, 14]. Detect identifies the secretory episodes on each time series with a *P* value equal to 0.01 (1%) for the nominal false-positive rate, as previously described [10, 13].

### Degree of concordance

The presence of significant concomitance between the secretory events of LH and kisspeptin was assessed by computing the specific concordance (SC) index, interposing various lags between each time series couplet under analysis [15]. The time series of each hormone (A and B) was converted into a “quantized” time series, where only the first sample (onset) of each detected peak was taken to represent the occurrence of that peak [15]. The quantized data series was then matched, and quantized values for hormone A were compared to the corresponding values for hormone B. The presence or absence of an event (onset) in either or both series was counted and the SC index was then computed. A spectrum of SC values was constructed by sliding the two series, interposing integral multiples of the sampling interval as the lag. The “0” point represents the first event (peak) from which the two temporal series are matched and then slided interposing then various lags of time (three time lags before and three time lag after, that is 30 min before or after). The presence of a positive lag time indicates that an event in A preceded the secretion event in B (15).

SC spectra were evaluated for each subject and the mean SCs over each group of patients were calculated at each lag time, obtaining a mean SC spectrum. The location of the maximum of the mean SCs for each group of women was also noted. Monte Carlo simulations were then performed to study the frequency distribution of the SC index under the null hypothesis of random concordance, as previously described [15]. For each of the two pairs of clinical data for each subject, 500 simulated pairs of simulated series were created and frequency distributions of SC obtained. An SC value above the 95% percentile of the frequency distribution generated for that individual or group of subjects resulted in rejection of the null hypothesis at the *P* < 0.05 confidence level [15].

### Statistical analysis

Data are expressed as mean ± SEM. We tested data for significant differences between groups, after analysis of variance (one-way ANOVA), using Student’s *t*-test for paired data. Pearson’s index was computed to evaluate correlation coefficients between groups. A *p* level < 0.05 was considered significant.

## Results

The characteristics of the patients included in this study are summarized in Table 1. Considering the variability between the two sample groups (menstrual intervals greater or less than 45 days), no differences were observed between hormonal parameters (Table 1, panel B and C).

**Table 1:** The hormonal and metabolic characteristics of the patients included in this study

	BMI	Glucose (mg/dl)	Insulin (µU/ml)	LH (mIU/ml)	FSH (mIU/ml)	LH/FSH	PRL (ng/ml)	E2 (pg/ml)	T (ng/ml)	FTI	Cortisol (µg/l)	DHEAS (µg/ml)	17OHP (ng/ml)	17KS (mg/24 h)	HOMA index	SHBG (ng/ml)
All PCOS under study(mean ± SEM)																
PCOS *(n*=71)																
	25.6 ± 0.3	88.2 ± 0.7	11.2 ± 0.5	12.3 ± 0.6	6.2 ± 0.1	2.1 ± 0.1	10.5 ± 0.4	56.3 ± 2.2	0.6 ± 0.04	19.2 ± 1.8	472.8 ± 17.8	0.9 ± 0.04	1.4 ± 0.08	11.3 ± 0.4	2.4 ± 0.1	42.6 ± 1.9
PCOS with menstrual cycle < 45 days (mean ± SEM)																
PCOS (*n*=30)																
	25.7 ± 0.2	88.1 ± 1.3	10.9 ± 1.0	12.6 ± 0.9	6.2 ± 0.2	2.0 ± 0.1	10.4 ± 0.7	59.0 ± 0.3	0.6 ± 0.07	16.8 ± 2.0	484.2 ± 27.5	0.9 ± 0.07	1.4 ± 0.1	11.5 ± 0.6	2.4 ± 0.2	45.2 ± 3
PCOS with menstrual cycle > 45 days (mean ± SEM)																
PCOS (*n*=41)																
	25.8 ± 0.3	88.2 ± 0.9	11.4 ± 0.7	13.2 ± 0.8	6.2 ± 0.2	2.2 ± 0.1	10.6 ± 0.6	54.3 ± 3.0	0.6 ± 0.05	20.9 ± 2.8	464.4 ± 23.8	0.97 ± 0.05	1.4 ± 0.1	11.1 ± 0.5	2.5 ± 0.1	40.6 ± 2.5

### Kisspeptin and LH pulsatile releases

When analyzing the time series of kisspeptin and LH, the Detect algorithm demonstrated the presence of spontaneous episodic events of LH and kisspeptin, both with a distinct pulsatile release pattern during the 2 h pulsatility study (Table 2). Interestingly, the group of PCOS patients with oligomenorrhea (i.e., menstrual interval greater than 45 days) showed a significantly higher kisspeptin pulse frequency as well as integrated serum concentrations than the eumenorrheic group of PCOS subjects (Table 2).

**Table 2:** Integrated plasma concentrations and pulse frequency for kisspeptin and LH

LH integrated mean (mIU/mL)	LH peaks/2 h	Kisspeptin integrated mean (ng/ml)	Kisspeptin peaks/2 h
A—All PCOS (*n* = 71) (mean ± SEM)			
11.1 ± 0.4	2.5 ± 0.1	1.65 ± 0.1	2.6 ± 0.1
B—PCOS with menstrual cycle < 45 days (*n* = 30) (mean ± SEM)			
10.6 ± 0.8	2.4 ± 0.1	1.12 ± 0.08	2.3 ± 0.1
C—PCOS with menstrual cycle > 45 days (*n* = 41) (mean ± SEM)			
11.5 ± 0.5	2.6 ± 0.1	2.0 ± 0.2 ***	2.8 ± 0.1*

*p < 0.04 and ***p < 0.0000004 versus PCOS with menstrual cycle < 45 days

**Fig. 1:**
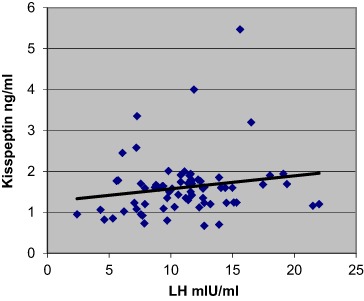
Correlation between mean plasma concentrations of LH and kisspeptin across all sample groups (*n* = 71), *p* < 0.001 (*r* = 0.36)

**Fig. 2:**
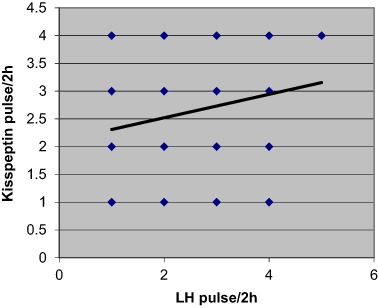
Correlation between mean pulse frequency over 2 h of LH and kisspeptin across all sample groups (*n* = 71). *P* < 0.05 (*r* = 0.22)

**Fig. 3:**
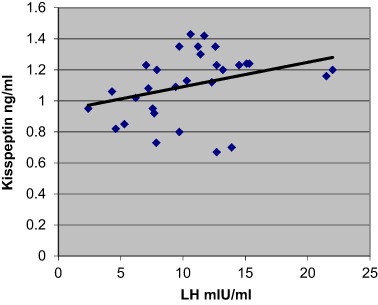
Correlation between mean plasma concentrations of LH and kisspeptin in the eumenorrheic PCOS patients (*n* = 30). *p* < 0.001 (*r* = 0.55)

Additionally, the data revealed a significant positive correlation between kisspeptin and LH concentrations, *p* < 0.001, (Fig. 1) and between LH and kisspeptin pulse frequencies across the whole group of PCOS patients, *p* < 0.001, (*n* = 71) (Fig. 2). However, when PCOS patients were subdivided into two groups, only eumenorrheic PCOS subjects demonstrated such significant correlation (*p* < 0.001)(Fig. 3). No correlation was observed for oligomenorrheic subjects (data not shown).

### Temporal coupling between kisspeptin and LH pulses

**Fig. 4:**
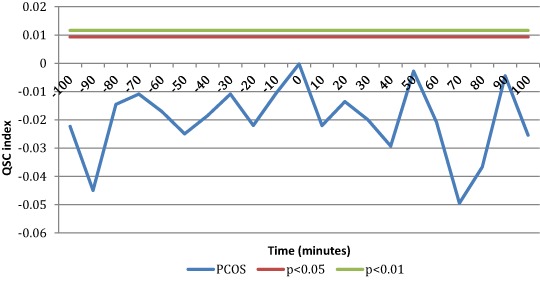
SC index computed for Kisspeptin and LH pulses across all sample groups (*n* = 71). As it can be seen no significancy was reached by SC index at any of the lag of time interval studied. This means no significant temporal coupling between kisspeptin and LH pulses

When temporal coupling between kisspeptin and LH pulses was computed, SC index was used to detect the presence of temporal coupling between LH and kisspeptin secretory events. As stated above, SC index is computed using occurrence of the secretory events rather than serum concentrations. When considering all PCOS patients, SC index did not reveal a significant degree of concordance between kisspeptin and LH pulses (Fig. 4). However, a complete different physiological setting was disclosed when subdividing the sample groups according to the menstrual interval length (i.e., eumenorrheic and oligomenorrheic patients).

**Fig. 5:**
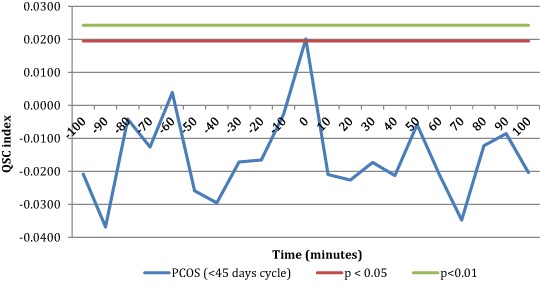
SC index between kisspeptin and LH pulses was significant at time 0 in the eumenorrheic PCOS group (*n* = 30)

Eumenorrheic PCOS, with a menstrual interval less than 45 days, showed a significant SC index at time 0, that is kisspeptin secretory events occur simultaneously with LH pulses, with no time lag (Fig. 5), suggesting that kisspeptin and LH are co-secreted. Conversely, SC index was not significant in the oligomenorrheic PCOS group with menstrual intervals greater than 45 days (Fig. 5). Practically no significant coupling exists between kisspeptin and LH secretory peaks in PCOS patients with oligomenorrhea.

### Correlations among hormonal and clinical parameters

**Fig. 6:**
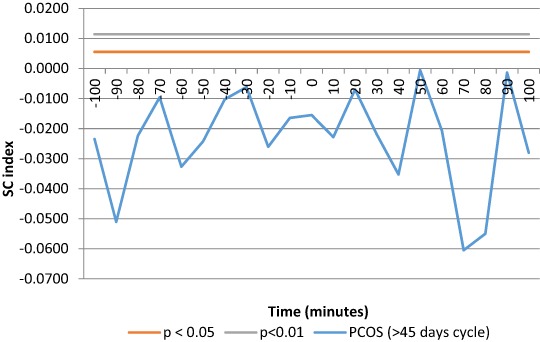
SC index between kisspeptin and LH pulses was not significant at any lag of time in oligomenorrheic PCOS group (*n* = 41)

A correlation analysis of biochemical and clinical indices was calculated on all patients in the study (Fig. 6). Supplemental Table 1 shows the Pearson’s coefficients for hormonal parameters of all PCOS patients. Kisspeptin mean serum concentrations did not correlate with LH and LH/FSH ratios in eumenorrheic patients (supplemental Table 2), while a significant correlation was observed in oligomenorrheic subjects (supplemental Table 3).

BMI and HOMA indices correlated with the majority of androgen and insulin serum levels across the both sample groups of PCOS patients (supplemental Table 1). When patients were subdivided according to the intermenstrual interval, the eumenorrheic group showed a correlation between BMI and insulin and HOMA index but not between BMI and T and FTI (supplemental Table 2). HOMA index strongly correlated with FTI and insulin (supplemental Table 2). Conversely, the oligomenorrheic group showed a correlation between BMI and androgens (T, FTI), insulin and HOMA index; HOMA index showed a strong correlation between FTI and insulin, similar to that of the eumenorrheic group (supplemental Table 3). Kisspeptin serum levels correlated with LH and the LH:FSH ratio only in the oligomenorrheic group (supplemental Table 3).

## Discussion

This observational study confirms the temporal coupling between kisspeptin and LH pulsatile releases and supports the hypothesis that PCOS reproductive impairment might be related and/or partly induced by an abnormal modulatory/regulatory role of kisspeptin on GnRH secretion from hypothalamic neurons. In fact the clinical aspect of oligomenorrhea occurs in those PCOS patients who did not revealed the coupling of kisspeptin with LH secretory episodes. Conversely eumenorrheic PCOS patients still show such coupling similarly to healthy subjects not affected by PCOS [10].

It is commonly understood that kisspeptin secretion is a key regulator of GnRH and LH pulsatile release and that abnormal LH concentration is a primary hormonal characteristic of PCOS [3]. Our previous report showed that young eumenorrheic females display spontaneous pulsatile release of kisspeptin and these secretory pulses are temporally coupled in the same moment, no lag of time, with LH secretory pulses [10]. Evidence of simultaneous secretion of the two hormones corroborates previous studies that demonstrate exogenous administration of kisspeptin triggers LH secretion [2]. In fact Narayanaswamy et. al [3] demonstrated that infusing subcutaneous kisspeptin-54 lead to an increase in LH serum levels, which correlated with an increase in serum estradiol concentrations. Such data clearly support that kisspeptin is a central neuroendocrine modulator of the human reproductive system.

Our present data provide compelling evidence that in PCOS patients spontaneous kisspeptin episodic secretion is coupled with LH pulses only when PCOS did not show oligomenorrhea, similar what previously observed during the follicular phase in eumenorrheic healthy women [10].

Indeed, when PCOS subjects were divided according to the clinical presence or absence of oligomenorrhea, significant differences were found. Both kisspeptin integrated serum concentrations and pulse frequencies were higher in oligomenorrheic than in eumenorrheic PCOS patients. More relevant, only eumenorrheic PCOS patients showed significant temportal coupling of kisspeptin and LH secretory episodes at time “0,” that is both kisspeptin and LH pulses occurred at the same moment, an observation perfectly similar to what previously reported for healthy eumenorrheic subjects [10]. Such similarity occurred also for both kisspeptin and LH concentrations and pulse frequency [10].

On the contrary, oligomenorrheic PCOS patients (menstrual interval > 45 days) did not show any temporal coupling between kisspeptin and LH secretory episodes.

In addition our study demonstrated that both kisspeptin serum concentrations and pulse frequencies were higher in oligomenorrheic than in eumenorrheic PCOS, as previously reported [16]. Based on these findings, PCOS reproductive dysfunction in oligomenorrheic patients might be considered to be related, at least in part, to an impaired kisspeptin regulation of GnRH discharge. In fact both the higher kisspeptin concentrations and the pulse frequency may be the expression of an impaired neuro-endocrine control of the hypothalamic nuclei, such as those secreting kisspeptin, thus altering the kisspeptin secretory episodes in terms of frequency and amplitide of the pulses and later affecting GnRH discharge, with the lack of concordance between kisspeptin with LH pulses.

According to our knowledge this study is the first to disclose the potential presence of an impaired linkage between pulsatile kisspeptin and LH secretions in PCOS, providing new insight to the pathophysiology of hypothalamo-pituitary functioning in this syndrome.

The present results are in agreement with previous studies of Chen et al. [17], Jeon et al. [18], and Yilmaz et al. [19], who reported elevated mean serum kisspeptin levels in PCOS women and Ozay et al. [20] who reported the positive correlations between kisspeptin, LH and leptin levels in PCOS patients. None of the previous studies took in consideration the clinical aspect of the intermenstrual intervals of their PCOS patients. Other authors reported lower kisspeptin serumlevels in overweight or obese PCOS women [16]. However, these studies measured only kisspeptin concentration in basaline conditions while we performed a rather extensive study considering the integrated mean over a pulsatility study, which is more indicative not only of the kisspeptin serum concentrations but also of changes of kisspeptin serum levels due to the presence of the endocrine spontaneous secretory episodes, as previously supported [13, 21].

Furthermore, murine studies of PCOS model confirm both higher kisspeptin secreting activity and neuronal density in the hypothalamus [21, 22]. Osuka et al. [23] generated two rat models of PCOS using two different androgen administration regimens. Exposure to dihydrotestosterone (DHT) led to the alteration of kisspeptin immunoreactivity, which led to different gonadotropin levels and ovarian morphologies. According to this model an excessive prenatal androgen exposure may cause higher kisspeptin levels in the arcuate nucleus (ARC) of the hypothalamus, which may result in a PCOS phenotype (normal body weight and higher serum LH levels).

It is obvious that animal models cannot be generalized and completely applied to humans but, in any case offer a putative pathophysiologycal model of PCOS that might facilitate a better understanding of the underlying mechanism that might lead to PCOS in humans. It cannot be excluded that the combination of ante/postnatal factors with ante/postnatal metabolic aspects may play a role in altering the activity of kisspeptin-secreting neurons, thus leading to the impaired GnRH-induced LH secretion.

Interestingly, while kisspeptin pulse frequency is increased in oligomenorrheic subjects, GnRH-induced LH discharge is unchanged as suggested by the similar LH pulse frequency of both eumenorrheic PCOS patients. However, the LH pulse frequency observed in both groups of PCOS patients were higher than in healthy controls [10]. On the contrary only eumenorrheic PCOS showed a kisspeptin episodic discharge similar to controls [10]. Among oligomenorrheic PCOS there was a higher kisspeptin pulse frequency than healthy controls [10].

The fact that oligomenorrheic PCOS shows higher kisspeptin pulse frequency but similar LH pulse frequency than eumenorrheic PCOS, let us infer that, as putative causal factor, also a neuroendocrine impairment might occur at the level of kisspeptin receptors and/or signal transduction on GnRH secreting neurons. Moreover, this rationale supports the possibility that oligomenrrheic PCOS patients are the physiopathological progression of the eumenorrheic patients.

According to what has been discussed, it seems plausible that the higher LH and kisspeptin pulse frequencies and serum concentrations observed in PCOS patients than those reported in healthy subjects [10] are due to the loss of a normal homeostatic equilibrium at the kisspeptin-GnRH secreting neuron level. Moreover the subtle but relevant differences inside the PCOS patients group (according to the eu-or oligomenorrheic condition) might reflect the deteriorating of the kisspeptin-GnRH axis functioning, thus leading to an impaired neuroendocrine function of the reproductive axis, as previously reported [24], though those with regular ovulatory cycles (and intermenstrual interval < 45 days) still show normal LH pulsatile patterns both in terms of amplitude and frequency [25], as our oligomenorrheic patients.

In concusion, our study demonstrated the presence of a distinct kisspeptin episodic secretion in PCOS patients and for the first time reported the abnormal temporal coupling between endogenous spontaneous kisspeptin and LH secretory episodes [26].

These data support the hypothesis that neuroendocrine impairments in PCOS affect the coupling of kisspeptin with LH pulses and potentially worsen as the disease progresses, becoming unequivocally evident among oligomenorrheic PCOS patients.

## Electronic supplementary material

Supplemental Figure

Supplemental Table 1

Supplemental Table 2

Supplemental Table 3

## References

[1] Skorupskaite K, George JT, Anderson RA (2014). The kisspeptin-GnRH pathway in human reproductive health and disease. Hum. Reprod. Update.

[2] Young J, George JT, Tello JA, Francou B, Bouligand J, Guiochon-Mantel A (2013). Kisspeptin restores pulsatile LH secretion in patients with neurokinin B signaling deficiencies: physiological, pathophysiological and therapeutic implications. Neuroendocrinology.

[3] Narayanaswamy S, Jayasena CN, Ng N, Ratnasabapathy R, Prague JK, Papadopoulou D (2016). Subcutaneous infusion of kisspeptin-54 stimulates gonadotrophin release in women and the response correlates with basal oestradiol levels. Clin. Endocrinol. (Oxf.)..

[4] Herbison AE (2008). Estrogen positive feedback to gonadotropin-releasing hormone (GnRH) neurons in the rodent: the case for the rostral periventricular area of the third ventricle (RP3V). Brain Res. Rev..

[5] Hrabovszky E (2014). Neuroanatomy of the human hypothalamic kisspeptin system. Neuroendocrinology.

[6] Genazzani AD, Luisi M, Malavasi B, Strucchi C, Luisi S, Casarosa E (2002). Pulsatile secretory characteristics of allopregnanolone, a neuroactive steroid, during the menstrual cycle and in amenorrheic subjects. Eur. J. Endocrinol..

[7] Genazzani AD, Petraglia F, Benatti R, Montanini V, Algeri I, Volpe A (1991). Luteinizing hormone (LH) secretory burst duration is independent from LH, prolactin, or gonadal steroid levels in amenorrheic women. J. Clin. Endocrinol. Metab..

[8] Genazzani AD, Petraglia F, Volpogni C, d’Ambrogio G, Facchinetti F, Genazzani AR (1993). FSH secretory pattern and degree of concordance with LH in amenorrheic, fertile and postmenopausal women. Am. J. Physiol..

[9] Meczekalski B, Genazzani AR, Genazzani AD, Warenik-Szymankiewicz A, Luisi M (2006). Clinical evaluation of patients with weight-loss related amenorrhea: neuropeptide Y and luteinizing hormone pulsatility. Gynecol. Endocrinol..

[10] Meczekalski B, Katulski K, Podfigurna-Stopa A, Czyzyk A, Genazzani AD (2016). Spontaneous endogenous pulsatile release of kisspeptin is temporally coupled with luteinizing hormone in healthy women. Fertil. Steril..

[11] Genazzani AD, Ricchieri F, Lanzoni C (2010). Use of metformin in the treatment of polycystic ovary syndrome. Women’s Health (Lond.).

[12] Henderson M, Rabasa-Lhoret R, Bastard JP, Chiasson JL, Baillargeon JP, Hanley JA (2011). Measuring insulin sensitivity in youth: how do the different indices compare with the gold-standard method?. Diabetes Metab..

[13] Oerter KE, Guardabasso V, Rodbard D (1986). Detection and characterization of peaks and estimation of instantaneous secretory rate for episodic pulsatile hormone secretion. Comput. Biomed. Res..

[14] Genazzani AD, Rodbard D (1991). Use of receiver operating characteristic curve to evaluate sensitivity, specificity, and accuracy of methods for detection of peaks in hormone time series. Acta Endocrinol. (Copenh).

[15] Guardabasso V, Genazzani AD, Veldhuis JD, Rodbard D (1991). Objective assessment of concordance of secretory events in two endocrine time series. Acta Endocrinol..

[16] Panidis D, Rousso D, Koliakos G, Kourtis A, Katsikis I, Farmakiotis D (2006). Plasma metastin levels are negatively correlated with insulin resistance and free androgens in women with polycystic ovary syndrome. Fertil. Steril..

[17] Chen X, Mo Y, Li Y, Yang D (2010). Increased plasma metastin levels in adolescent women with polycystic ovary syndrome. Eur. J. Obstet. Gynecol. Reprod. Biol..

[18] Jeon YE, Lee KE, Jung JA (2013). Kisspeptin, leptin, and retinol-binding protein 4 in women with polycystic ovary syndrome. Gynecol. Obstet. Invest..

[19] Yilmaz SA, Kerimoglu OS, Pekin AT, Incesu F, Dogan NU, Celik C, Unlu A (2014). Metastin levels in relation with hormonal and metabolic profile in patients with polycystic ovary syndrome. Eur. J. Obstet. Gynecol. Reprod. Biol..

[20] Ozay O, Ozay AC, Acar B, Cagliyan E, Seçil M, Küme T (2016). Role of kisspeptin in polycystic ovary syndrome (PCOS). Gynecol. Endocrinol..

[21] Kondo M, Osuka S, Iwase A, Nakahara T, Saito A, Bayasula (2016). Increase of kisspeptin-positive cells in the hypothalamus of a rat model of polycystic ovary syndrome. Metab. Brain Dis..

[22] Matsuzaki T, Tungalagsuvd A, Iwasa T, Munkhzaya M, Yanagihara R, Tokui T (2017). Kisspeptin mRNA expression is increased in the posterior hypothalamus in the rat model of polycystic ovary syndrome. Endocr. J..

[23] Osuka S, Iwase A, Nakahara T, Kondo M, Saito A, Bayasula (2017). Kisspeptin in the hypothalamus of two rat models of polycystic ovary syndrome. Endocrinology.

[24] Patel K, Coffler MS, Dahan MH, Malcom PJ, Deutsch R, Chang RJ (2004). Relationship of GnRH-stimulated LH release to episodic LH secretion and baseline endocrine-metabolic measures in women with polycystic ovary syn- drome. Clin. Endocrinol. (Oxf.)..

[25] Adams JM, Taylor AE, Crowley WF, Hall JE (2004). Polycystic ovarian morphology with regular ovulatory cycles: insights into the pathophysiology of polycystic ovarian syndrome. J. Clin. Endocrinol. Metab..

[26] Meczekalski B, Podfigurna-Stopa A, Genazzani AR (2011). Why kisspeptin is such important for reproduction?. Gynecol. Endocrinol..

