# Supplementary material for: Kisspeptin and LH pulsatile temporal coupling in PCOS patients
Source: Endocrine. 2018 May 4;61(1):149–57. doi: 10.1007/s12020-018-1609-1 (PMC5997113; doi:10.1007/s12020-018-1609-1)
Supplement: Supplementary file 1 — Supplemental Figure [file 12020_2018_1609_MOESM1_ESM.docx]

Supplemental Fig 3. Correlation between mean plasma concentrations of LH and kisspeptin in the eumenorrheic PCOS patients (n=30). p<0.001 (r=0.55)
