# Supplementary material for: Kisspeptin and LH pulsatile temporal coupling in PCOS patients
Source: Endocrine. 2018 May 4;61(1):149–57. doi: 10.1007/s12020-018-1609-1 (PMC5997113; doi:10.1007/s12020-018-1609-1)
Supplement: Supplementary file 4 — Supplemental Table 3 [file 12020_2018_1609_MOESM4_ESM.doc]

**Supplemental Table 3.** The Pearson’s coefficients for hormonal parameters across oligomenorrheic PCOS patients.

| PCOS > 45 days  N=41 | BMI | LH  mIU/ml | LH/FSH | Cortisol  µg/l | 17OHP  ng/ml | T  ng/ml | FTI | Insulin µU/ml  time 0’ | Insulin µU/ml time 60’ | Kisspeptin  ng/ml | Kiss pulses/2h | HOMA-R |
| --- | --- | --- | --- | --- | --- | --- | --- | --- | --- | --- | --- | --- |
| BMI |  |  |  |  |  | 0.01 | 0.0001 | 0.01 |  |  |  |  |
| LH |  |  | 0.000001 |  |  |  |  |  |  | 0.001 |  |  |
| LH/FSH |  | 0.000001 |  |  |  | 0.01 |  |  |  | 0.05 |  |  |
| Cortisol |  |  |  |  | 0.05 |  |  |  |  |  |  |  |
| 17OHP |  |  |  | 0.05 |  | 0.001 | 0.05 | 0.05 |  |  |  | 0.05 |
| T | 0.01 |  | 0.01 |  | 0.001 |  | 0.00001 |  |  |  |  |  |
| FTI | 0.0001 |  |  |  | 0.05 | 0.00001 |  |  |  |  |  |  |
| Insulin time 0’ | 0.01 |  |  |  | 0.05 |  |  |  |  |  |  |  |
| Insulin time 60’ |  |  |  |  |  |  |  |  |  |  |  |  |
| Kisspeptin |  | 0.001 | 0.05 |  |  |  |  |  |  |  |  |  |
| Kiss pulses/2h |  |  |  |  |  |  |  |  |  |  |  |  |
| HOMA-R |  |  |  |  | 0.05 |  |  |  |  |  |  |  |
|  |  |  |  |  |  |  |  |  |  |  |  |  |
